# Expert Credibility and Sentiment in Infodemiology of Hydroxychloroquine’s Efficacy on Cable News Programs: Empirical Analysis

**DOI:** 10.2196/45392

**Published:** 2023-06-27

**Authors:** Dobin Yim, Jiban Khuntia, Elliot King, Matthew Treskon, Panagis Galiatsatos

**Affiliations:** 1 Loyola University Baltimore, MD United States; 2 Health Administration Research Consortium University of Colorado Denver Denver, CO United States; 3 Johns Hopkins University Baltimore, MD United States; 4 Johns Hopkins School of Medicine Baltimore, MD United States

**Keywords:** source credibility, infodemic, infoveillance, broadcasting, cable television, COVID-19

## Abstract

**Background:**

Infodemic exacerbates public health concerns by disseminating unreliable and false scientific facts to a population. During the COVID-19 pandemic, the efficacy of hydroxychloroquine as a therapeutic solution emerged as a challenge to public health communication. Internet and social media spread information about hydroxychloroquine, whereas cable television was a vital source. To exemplify, experts discussed in cable television broadcasts about hydroxychloroquine for treating COVID-19. However, how the experts’ comments influenced airtime allocation on cable television to help in public health communication, either during COVID-10 or at other times, is not understood.

**Objective:**

This study aimed to examine how 3 factors, that is, the credibility of experts as doctors (DOCTOREXPERT), the credibility of government representatives (GOVTEXPERT), and the sentiments (SENTIMENT) expressed in discussions and comments, influence the allocation of airtime (AIRTIME) in cable television broadcasts. SENTIMENT pertains to the information credibility conveyed through the tone and language of experts’ comments during cable television broadcasts, in contrast to the individual credibility of the doctor or government representatives because of the degree or affiliations.

**Methods:**

We collected transcriptions of relevant hydroxychloroquine-related broadcasts on cable television between March 2020 and October 2020. We coded the experts as DOCTOREXPERT or GOVTEXPERT using publicly available data. To determine the sentiments expressed in the broadcasts, we used a machine learning algorithm to code them as POSITIVE, NEGATIVE, NEUTRAL, or MIXED sentiments.

**Results:**

The analysis revealed a counterintuitive association between the expertise of doctors (DOCTOREXPERT) and the allocation of airtime, with doctor experts receiving less airtime (*P<.*001) than the nonexperts in a base model. A more nuanced interaction model suggested that government experts with a doctorate degree received even less airtime (*P=.*03*)* compared with nonexperts*.* Sentiments expressed during the broadcasts played a significant role in airtime allocation, particularly for their direct effects on airtime allocation, more so for NEGATIVE (*P<.*001), NEUTRAL *(P<.*001), and MIXED *(P=.*03) sentiments. Only government experts expressing POSITIVE sentiments during the broadcast received a more extended airtime (*P<.*001) than nonexperts*.* Furthermore, NEGATIVE sentiments in the broadcasts were associated with less airtime both for DOCTOREXPERT (*P<.*001) and GOVTEXPERT (*P<.*001).

**Conclusions:**

Source credibility plays a crucial role in infodemics by ensuring the accuracy and trustworthiness of the information communicated to audiences. However, cable television media may prioritize likeability over credibility, potentially hindering this goal. Surprisingly, the findings of our study suggest that doctors did not get good airtime on hydroxychloroquine-related discussions on cable television. In contrast, government experts as sources received more airtime on hydroxychloroquine-related discussions. Doctors presenting facts with negative sentiments may not help them gain airtime. Conversely, government experts expressing positive sentiments during broadcasts may have better airtime than nonexperts. These findings have implications on the role of source credibility in public health communications.

## Introduction

### Background

An infodemic is an expression that blends the words information and epidemic. An infodemic occurs when accurate or inaccurate information rapidly spreads everywhere; the overabundance makes it difficult for people to find trustworthy sources and reliable guidance when needed [1,2]. The dispersion of facts and rumors often bleed into each other in an infodemic, as the information spreads concerns and fears among the public [2,3]. Subsequently, it becomes challenging to learn the correct and essential information.

Prior studies on health information retrieval, spread, and dissemination in flu contexts have asserted infodemiology as a vital area of research needing more attention to explore deeper nuanced mechanisms of health communications [3,4]. Combating infodemics involves awareness, literacy, fact-checking, monitoring (infoveillance), and the nondistortion of facts [2]. More studies would help design and monitor accurate health communication strategies that can disseminate scientific facts to inform public health and policy [5-7].

Early work in media and information management has suggested that people are more likely to be persuaded when a source presents itself as credible while disseminating information [8-10]. A relevant concept of medium credibility would evaluate the medium through which the message is delivered and the characteristics of the message source, such as how social media or newspapers influence persuasion [11]. News and media channels must identify areas where there is a knowledge translation gap between best evidence (what some experts know) and practice (what most people do or believe), as well as markers for “high-quality” information to curb the spread of misinformation [3].

Research must inform how scientific credibility in communication helps manage the spread of information. In this context, source credibility is a concept that focuses on the origin of the fact, message, or information. The source may refer to the government, a nonprofit agency, or a corporation. News and media agencies ratify information through experts from scientific institutions, agencies, or academia to provide credibility [12]. The audience may consider these experts as primary sources. Thus, it is crucial to understand how the source credibility affects the expert-ratified information dissemination during infodemics, which is the objective of this study.

### Infodemic During COVID-19

The issue of infodemic was quite apparent during the COVID-19 pandemic, with several pieces of information spreading swiftly; the accuracy of the fact-checking was questionable [13]. In February 2020, as the gravity of the threat posed by COVID-19 came to be recognized internationally, the Director-General of the World Health Organization declared that the world must fight not only the epidemic but also an infodemic [14].

The rapid spread of COVID-19 raised many difficult questions, including what the origin of the virus was, how transmissible it was, how lethal it would be, what mitigation measures might be required to minimize its impact, and how effective the potential treatments and therapeutic drugs were. Given the array of questions to which there were no known answers, the number of COVID-19 cases skyrocketed, and therefore the consumption of information about the pandemic soared [15]. Several studies revealed that the COVID-19–related content found on many social media platforms was inconsistent and unreliable [16-18], leading to infodemic challenges during this period of uncertainty.

### Overview of Hydroxychloroquine in Public Discourse

The spread of information about hydroxychloroquine in public discourse during COVID-19 is an exemplary infodemic. The idea that hydroxychloroquine could be an effective therapeutic for COVID-19 began circulating in China in January 2020. Subsequently, it spread through social media in Nigeria; Vietnam; France; and ultimately in the United States in early March when Paul Sperry, a conservative author, tweeted it on March 9. On March 13, investor James Toldano tweeted a link to a Google Document he had coauthored with Gregory Rigano, a lawyer, touting the benefits of hydroxychloroquine.

In March 2020, the idea that hydroxychloroquine could be effective against COVID-19 was first raised publicly with subsequent infodemics [19]. On March 16, Lara Ingraham discussed the drug with Dr Anthony Fauci on her show, and on March 18, Rigano was interviewed on The Tucker Carlson Show; both the shows were broadcasted on FOX News. On the same day, a reporter asked about the potential of hydroxychloroquine as a therapeutic for COVID-19 at a White House briefing. On March 19, at another White House briefing, President Trump touted the drug as a “potential game changer.” On March 28, the Food and Drug Administration issued an emergency use authorization, empowering doctors to prescribe hydroxychloroquine to fight COVID-19. Approximately 1 month later, on April 24, the Food and Drug Administration cautioned against using hydroxychloroquine as a treatment for COVID-19, and on June 15, it rescinded the emergency use authorization. In addition, subsequent clinical trials established hydroxychloroquine as an ineffective treatment for COVID-19. The National Institutes of Health stated that hydroxychloroquine was ineffective for COVID-19 in November 2020.

The public interest sparked by the media coverage was evident. The number of prescriptions for using hydroxychloroquine increased from approximately 30,000 in February 2020 to >220,000 in March. However, the number of prescriptions reduced to approximately 100,000 in April and 35,000 in May [20]. There is some evidence that the publicity given to hydroxychloroquine as a therapeutic for COVID-19 led to shortages of the drug for patients who need it for other reasons [21]. On November 9, 2020, the National Institutes of Health issued a press release based on a study that appeared the same day in the Journal of the American Medical Association, stating that hydroxychloroquine does not provide a clinical benefit to adults hospitalized with COVID-19 [22].

### Research Gap and Questions

Prior research points to the role of social and other media in scientific credibility and health communication contexts [5-7]. The role of source credibility as a persuasive element remains relatively unexplored [8-10]. More specifically, given the consequential nature of the context of broadcasting information about hydroxychloroquine in public discourse during COVID-19 [23,24], misleading information spread [25,26] points to the need to conduct research exploring source credibility as an element in health communications.

Existing literature that explores hydroxychloroquine in public discourse by using social and other media is sparse. A prior study has identified and characterized scientific authority–related discussions about hydroxychloroquine, alluding to medical experts’ credibility aspect of sources [27]. Other studies have explored how emotional-moral words correlate with a higher likelihood of being retweeted, how emotions are essential in making content contagious on social media [27-30], and how moral emotions shaped information spread on Twitter and other media about hydroxychloroquine as a solution to COVID-19 [29,30].

News broadcasts played a substantial role in disseminating information about hydroxychloroquine. Broadcasts used experts from institutions, agencies, or universities, who may have been perceived as the primary source by the audience [12]. It is crucial to understand whether this expert-ratified information was helpful. However, no study provides insights into how expert opinions during the broadcast provided credibility. To address this research gap in the context of hydroxychloroquine in public discourse during COVID-19, we asked, (1) Do credible information sources influence the infodemic process? If so, how? and (2) Which attributes of the source credibility influence the dynamics of information spread?

### Study Road Map

This study examines how 3 factors—the credibility of experts as doctors (DOCTOREXPERT), the credibility of government representatives (GOVTEXPERT), and the sentiments (SENTIMENT) expressed in cable television discussion broadcasts—influence the allocation of airtime (AIRTIME) for hydroxychloroquine in public discourse during COVID-19. The data were collected from transcripts of cable television broadcasts and coded using machine learning algorithms. We used Tobit regression models to estimate the effect of experts’ credibility and sentiment on airtime. The implications of the findings of our analysis are discussed.

## Methods

### Sampling Period and Strategy

The study period spans from March 1, 2020, to November 30, 2020. The first mention of hydroxychloroquine on cable news was on March 1, 2020. We noted that the first mention of hydroxychloroquine as a potential treatment for COVID-19 symptoms in a tweet by Elon Musk with a link to a Google Document occurred on March 16, 2020. However, discussions regarding the potential use were present on social media earlier. The National Institutes of Health declared the drug ineffective against COVID-19 on November 30, 2020. The data collection and coding process for this study followed several steps: the identification of the days in which hydroxychloroquine was most discussed on 3 primary cable news networks from March 2020 to November 2020; collection of the broadcast videos; identification of the experts and collection of information about them, calculating the amount of airtime the medical experts on each network received during the discussion of hydroxychloroquine; and an assessment of sentiments expressed in their remarks.

### Data Collection Process

The study’s data set comes from Stanford Cable TV News Analyzer [31], which collects data from the Internet Archive for television data set that consists of >300,000 video recordings. A vital feature of the Stanford Cable TV News Analyzer augments the Internet Archive data set with a trend dashboard that helps to create a curated database of video segments from cable news, enabling us to conduct focused searches. One key feature of the analyzer is its keyword search query tool, which allows us to identify video segments where specific words are spoken by participants by using the transcript of the video as a reference. This functionality provides valuable insights into experts’ sentiments as expressed in the cable television broadcasts.

According to the Stanford Cable TV News Analyzer, a video segment is defined as an approximately 3-minute interval from a cable news show in which at least 1 panel expert mentions the keyword (eg, hydroxychloroquine) in the news transcript. The daily totals indicate the interest cable news networks had in hydroxychloroquine. The search query was performed at the “daily” level; thus, the daily aggregation unit generated a time trend chart to identify the peak periods of hydroxychloroquine-related discussions on the 3 US cable news networks. We defined peak periods as days in which the search results of hydroxychloroquine returned at least ≥20 video segments. We removed dates during which the total daily number of video segments aired was <20 to focus on the high-interest level periods, resulting in 565 unique video segments. Table 1 provides information on the broadcasts on key dates.

**Table 1:** Information about the broadcasts.

Dates	April 6	April 22	April 23	April 24	May 6	May 14	May 18	May 19	May 20	May 21	May 22	May 23	July 28	July 29	July 30	July 21	August 3	October 2
Total videos, n	29	43	37	43	22	33	25	56	38	29	32	29	28	51	24	24	27	24
Seconds per episode	6.62	5.58	5.35	4.05	1.99	4	11.52	11.04	5.84	2.28	6	4.34	6	6.35	3.5	3.75	4.44	2.05
Minutes per day	3.2	4	3.3	2.9	0.73	2.2	4.8	10.3	3.7	1.1	3.2	2.1	2.8	5.4	1.4	1.5	2	0.82

Once we identified the dates when cable news prominently featured discussions about hydroxychloroquine with panels of experts, we used the query tool by entering 2 variations of hydroxychloroquine, “Hydroxychloroquine” and “Hydroxy,” as the keywords. We added the names of 3 main cable news channels: “FOX, CNN, and MSNBC.” We also limited the search by adding the term “aired between March 1, 2020, and November 30, 2021.” The search was performed using a publicly available Python package on open-source GitHub Archives [32] to query the television archive database. We modified an original Python script (*get_news_identifiers.py*) to implement the search strategy for the videos that matched the key dates. We found 1147 videos, of which 425 (37.05%) were from Cable News Network (CNN), 357 (31.12%) were from FOX News, and 365 (31.82%) were from MSNBC cable networks. We then retrieved the full-text captioning of the videos using another script (ie, *scrape_archive_org.py*), returning HTML files as output, with captions demarcated to the minute. The script identifies and parses the text segment based on the start and end of the time stamps identified from the previous data-coding process. Then, we filtered a subset of these videos whose full text included the word “hydroxychloroquine” or “hydroxy.” Filtering for hydroxychloroquine yielded 585 videos (CNN: n=273, 46.7%; FOX News: n=117, 20%; and MSNBC: n=195, 33.3%). Upon final review, we removed 10 videos because they were duplicates, resulting in 575 videos.

### Experts’ Information in the Broadcasts

The sampled videos were then shared with coders that marked the expert speaker, comment start time, and comment end time. For each video segment identified during the peak period dates, we obtained the names and affiliations of the experts and measured the amount of airtime they received by marking the time stamps of their first and last appearances within the segment. A custom Python script extracted the text of the expert speaker to the nearest minute. Because the time marker of the transcript is at a 1-minute interval, the parsing procedures may include extraneous text, such as the host’s introduction of the expert in the output text. Although the added text by the host may introduce potential errors in extracting the expert’s core message, the nature of the content is related and relevant; thus, the validity of the analysis would remain intact.

The coding process involved a team of 3 researchers and 3 graduate students who analyzed each person featured in the video segment the show hosts interviewed. Typically, the identifying information about a person, such as their name, credentials, and affiliation, appeared at the bottom of the screen. The coders categorized a person as an expert if their credentials listed a terminal doctorate in medicine or a relevant scientific discipline such as microbiology or epidemiology. Otherwise, the person was coded as a nonexpert. If the video segment did not provide complete credentials and affiliations, the coders searched Google and LinkedIn to verify their expert status. Individuals whose incomplete information could not be verified were excluded from the data set. The coders deliberated on individuals who sounded knowledgeable to include or exclude in the experts’ categories, with the inclusion criteria that evaluating or providing expert inputs on hydroxychloroquine’s effectiveness as therapeutic for COVID-19-related symptoms requires a scientific or clinical understanding of its applicability as a new treatment alternative. We excluded politicians, lobbyists, lawyers, news contributors, correspondents, hosts, and political appointees holding administrative positions in organizations who did not have academic credentials or prior professional experience in the medical-related field.

We measured the amount of airtime received by experts by recording the start and end time stamps of the conversations between the news host and the experts. The duration of the host’s introduction was subtracted from the calculation. If the conversation involved multiple exchanges between the host and the expert, the total duration of the expert’s appearance was recorded. In cases where multiple experts were featured in the show, each expert’s contribution was captured separately. We addressed syntax and duration calculation errors in the samples and removed samples with missing data. In total, we identified 354 unique experts.

### Sentiment Analysis of the Samples Broadcasts

The entire corpus was processed using latent Dirichlet allocation-based topic modeling and an automated sentiment analysis program using Amazon Web Service Comprehend (AWSC). This cloud-based automated service uses machine learning to process the videos’ full text for sentiment analysis. This process involves training a classifier on a labeled data set to predict sentiment polarity, including positive, negative, and neutral categories. AWSC is similar to other commercial software applications such as Linguistic Inquiry and Word Count (LIWC) or NVivo and open-source programming languages such as Python and R, which provide sentiment classifier packages such as Natural Language Toolkit, Gensim, and topic modeling. These packages enable automatic tabulation and numerical calculation of sentiment scores for sentences, paragraphs, and documents. Typically, sentiment scores range from 0 (lowest) to 1 (highest) for discrete sentiment polarity or from −1 (negative) to 1 (positive) for a combined sentiment polarity scale. However, AWSC was preferred because of the ease and appropriateness of analyzing extensive text data, scalability, and the latest features while accurately identifying positive and negative sentiments in text.

Sentiment analysis approaches have been used in prior research to understand mediated and health-related content; for example, a study analyzed positive, negative, neutral, and ambiguous tones of tweets on e-cigarettes [33]. Studies have used computer-aided sentiment analysis to annotate sentiments’ directionality to better understand sentiments experienced following alcohol-induced blackouts [34] and on breast cancer social networks [35].

The sentiment analysis process follows a lexicon-based classification that categorizes each word in each text as positive, negative, or neutral based on a predefined dictionary. For instance, words such as “joy,” “happy,” and “excited” are classified as positive sentiment words, whereas “angry,” “scared,” and “sad” fall into the negative sentiment category. The number of words identified in each sentiment category or polarity can then be compared across different documents. The count of sentiment polarity occurrences in each document can be normalized on a standard scale or combined into a single scale, such as −1 to 1, where negative values indicate negative polarity, positive values denote positive sentiment, and 0 represents neutral sentiment. However, inferring document sentiment solely based on the relative occurrence of sentiment-embedded words can be challenging, especially in cases where such words are sparse, such as in academic manuscripts. A machine learning approach trains a model on a prelabeled data set of documents and their corresponding sentiment outcomes as discrete categories or numerical scores to derive a predictive sentiment classifier, overcoming the sparse-word challenge. This model can then predict the sentiment for a focal set of documents. To better understand what might drive the sentiment scores, we isolated the comments made by the experts and ranked them according to their sentiment scores. Examples of expert comments with high positive or negative sentiment scores are shown in Textbox 1.

**Examples of positive and negative sentiments in experts’ comments.**

**Examples of positive sentiment broadcast comments**

- “We continue to study the effectiveness of Hydroxychloroquine and other therapies in the treatment and prevention of the virus, and we will keep the American people fully informed of our fighting. Hydroxychloroquine is looking like it’s having some good results. i hope that would be a phenomenal thing but we have it right now.” [Mehmet Oz, FOX News on April 4, 2020; score: 0.982]
- “...Hydroxychloroquine that the doctor was talking about in test tubes and seems to be more effective against the virus, and this is the one that has been used more or less around the world. this is the one that the French looked at and had a pretty profound response...I’m very happy about the University of Minnesota is testing and studying this drug. The University of Washington is giving six patients, and what it looks like it’s coming out about this drug is it works better if it is used early in the process before the coronavirus covid-19 really takes on steam. so that’s what I am looking at.” [Marc Siegel, FOX News, on March 24, 2020; score: 0.983]

**Examples of negative sentiment broadcast comments**

- “Some compounds in a test tube appear to have an anti-viral capacity and are worthless in humans. A recent example of a compound like that is Hydroxychloroquine, which in vitro appeared to have antiviral capabilities but, tested in human beings, is worthless...you hear proponents of this people say I have seen it with my own eyes have incredible power, which you know is all well and good. it sounds great, and maybe that person actually believes it, but that is not actually how science works.” [Jonathan Reiner, Cable News Network (CNN), on August 17, 2020; score: 0.996]
- “I can’t prescribe Hydroxychloroquine for my lupus patients because so many other people have gotten prescriptions who don’t need them. you can see how the misinformation actually leads to pretty bad consequences for patients...it is pretty bad.” [Kavita Patel, MSNBC, on April 5, 2020; score: 0.975]
- “The American corporations...are globalist and they want to push a global agenda and make sure that when the time comes for China to be open to that they aren’t on the wrong side of China’s propaganda arm the Chinese government. That’s why they are allowing it. if people are telling people, Hydroxychloroquine doesn’t work. Saying that they will die if they take it. they are being allowed to get.” [Harmeet Dhillon, FOX News, on April 1, 2020; score: 0.973]

### Ethical Considerations

The data collected for this study were obtained from publicly available sources. The study did not involve any interaction with users. Therefore, ethical approval was not required for this study.

### Sample Statistics

Table 2 shows the descriptive statistics and pairwise correlations among the key variables used in this study. On average, experts were featured for 264.72 seconds per cable news show in which they appeared. Their statements expressed an average score of 0.16 for positive sentiments, 0.29 for negative sentiments, 0.33 for neutral sentiments, and 0.21 for mixed sentiments. Of the 565 video segments analyzed, 354 (62.7%) featured experts and 64 (11.3%) featured government affiliates. Of the 565 video segments analyzed, the largest number of monthly totals was aired in April, with 171 (30.3%) video segments, followed by 150 (26.6%) segments in May, 100 (17.7%) segments in March, 87 (15.4%) segments in August, 45 (8%) segments in July, and 12 (2.1%) segments in October.

**Table 2:** Summary statistics and pairwise correlations among key variables (number of observations=565).

Variables	Value, mean (SD)	Value, range	ln(AIRTIME)	DOCTOREXPERT	GOVTEXPERT	POSITIVE	NEGATIVE	NEUTRAL	MIXED	March	April	May	July	August	October
ln(AIRTIME)	5.13 (1.00)	1.39-8.15	1.00	—^a^	—	—	—	—	—	—	—	—	—	—	—
DOCTOREXPERT	0.63 (0.48)	0-1	−0.14	1.00	—	—	—	—	—	—	—	—	—	—	—
GOVTEXPERT	0.11 (0.32)	0-1	0.16	−0.14	1.00	—	—	—	—	—	—	—	—	—	—
POSITIVE	0.16 (0.18)	0-0.94	0.21	0.02	0.25	1.00	—	—	—	—	—	—	—	—	—
NEGATIVE	0.29 (0.23)	0-0.97	−0.07	−0.06	−0.16	−0.54	1.00	—	—	—	—	—	—	—	—
NEUTRAL	0.33 (0.21)	0-0.98	−0.16	−0.09	0.03	−0.05	−0.37	1.00	—	—	—	—	—	—	—
MIXED	0.21 (0.21)	0-0.93	0.05	0.13	−0.07	−0.22	−0.25	−0.55	1.00	—	—	—	—	—	—
March	0.18 (0.38)	0-1	0.16	−0.01	0.24	0.27	−0.30	0.08	0.01	−0.53	1.00	—	—	—	—
April	0.30 (0.46)	0-1	−0.01	0.12	−0.02	0.03	−0.04	0.03	−0.01	−0.39	−0.31	1.00	—	—	—
May	0.27 (0.44)	0-1	−0.11	−0.11	−0.18	−0.13	0.17	0.00	−0.07	−0.02	−0.28	−0.40	1.00	—	—
July	0.08 (0.27)	0-1	−0.20	0.02	0.08	−0.02	0.03	−0.06	0.05	0.32	−0.14	−0.19	−0.18	1.00	—
August	0.15 (0.36)	0-1	0.14	−0.01	−0.06	−0.18	0.19	−0.12	0.07	0.69	−0.20	−0.28	−0.26	−0.13	1.00
October	0.02 (0.14)	0-1	0.02	−0.06	−0.05	0.03	−0.06	0.08	−0.05	0.40	−0.07	−0.10	−0.09	−0.04	−0.06

^a^Not applicable.

### Study Variables

The unit of analysis is the expert’s appearance on a cable news show per video segment. The dependent variable in this study is AIRTIME. AIRTIME is measured by calculating the difference between the start and end time of a guest’s appearance on the cable news network’s show in seconds. The values were log transformed to mitigate the skewed distribution of airtime. Table 2 displays that, on average, AIRTIME is 5.13 or 264.72 seconds.

A total of 6 independent variables are of interest in this study. The first 2 are DOCTOREXPERT and GOVTEXPERT. The second set includes the 4 types of sentiments expressed in the broadcasts: POSITIVE, NEGATIVE, NEUTRAL, and MIXED. The independent variable, DOCTOREXPERT, identified a featured guest’s expertise on the subject matter because of the advanced doctorate degree and subsequent clinical practice involvements. If the featured guest had a degree in medicine or an advanced degree in a relevant scientific discipline such as microbiology or epidemiology, the variable was coded as 1 and otherwise as 0. The study sample featured an approximately equal distribution of experts (354/565, 62.7%) and nonexperts. The second independent variable, GOVTEXPERT, identified a featured guest’s affiliation with a government organization. An affiliation variable with other organizations, such as academic institutions, health organizations, news organizations, or private practice, was also considered. Only one affiliation type was associated with each featured guest, and the variable was coded as 1 for the affiliation and otherwise as 0. Of the various affiliations, only the government affiliation (64/565, 11.3%) was considered for this study, as other affiliations did not show any statistical significance to explain airtime. Together, these variables comprise a featured guest’s credibility in their expertise to report facts or opinions about hydroxychloroquine as a legitimate therapeutic for COVID-19.

The machine learning algorithm measured the 4 variables associated with sentiments expressed in featured guests’ statements. Each measurement scale ranged from 0 to 1, with 1 representing the highest level of sentiment expressed. In general, featured guests showed more sentiments in their statements, with combined sentiment scores of 0.67: POSITIVE, NEGATIVE, MIXED, and NEUTRAL sentiments scored 0.16, 0.29, 0.21, and 0.33, respectively.

### Statistical Analyses

The empirical model examined the relationship between experts’ credibility, experts’ sentiments expressed during broadcasts, and the airtime they received. The models included controls for months and days adjust for variations in the opportunities and interests of experts, and variation in the number of cable news appearances over time appearing on cable news networks to discuss hydroxychloroquine. Tobit regression was used that accounted for extreme airtime values at the upper and lower bounds, where some experts who should have received airtime did not appear on the show. The specified and estimated interaction models build on a base model that specify credibility variables’ direct effects on airtime. We then each one of the 3 highly correlated sentiment-dummy variables separately in regressions to avoid multicollinearity, improve the accuracy and comprehensiveness of the analysis, and draw comparable insights about each variable. We added dummy variables reflecting cable news channels to cluster the error variances that may arise from the repeated measures of cable news shows. Including time dummy variables and cable news clustering variables minimizes the bias associated with the model specification. Textbox 2 shows the interaction model specifications that were estimated, in which *i* denotes one broadcast as the unit of analysis:

**Interaction model with DOCTOREXPERT and GOVTEXPERT.**

- *1.1: ln(Airtime)i = β0 + β1DOCTOREXPERTi + β2GOVTEXPERTi + β3DOCTOREXPERTi × GOVTEXPERTi + Controlsi + εi*
- *1.2: ln(Airtime)i = β0 + β1DOCTOREXPERTi + β2GOVTEXPERTi + β3POSITIVEi + β4DOCTOREXPERTi × GOVTEXPERTi + β5DOCTOREXPERTi × POSITIVEi + β6GOVTEXPERTi × POSITIVEi + Controlsi + εi*
- *1.3: ln(Airtime)i = β0 + β1DOCTOREXPERTi + β2GOVTEXPERTi + β3NEGATIVEi + β4DOCTOREXPERTi × GOVTEXPERTi + β5DOCTOREXPERTi × NEGATIVEi + β6GOVTEXPERTi × NEGATIVEi + Controlsi + εi*
- *1.4: ln(Airtime)i = β0 + β1DOCTOREXPERTi + β2GOVTEXPERTi + β3NEUTRALi + β4DOCTOREXPERTi × GOVTEXPERTi + β5DOCTOREXPERTi × NEUTRALi + β6GOVTEXPERTi × NEUTRALi + Controlsi + εi*
- *1.5: ln(Airtime)i = β0 + β1DOCTOREXPERTi + β2GOVTEXPERTi + β3MIXEDi + β4DOCTOREXPERTi × GOVTEXPERTi + β5DOCTOREXPERTi × MIXEDi + β6GOVTEXPERTi × MIXEDi + Controlsi + εi*

## Results

### Overview

Cable television broadcasts used in this sample for hydroxychloroquine span approximately 5 to 265 seconds, with high participation of academic doctor experts (354/565, 62.7%) but fewer government experts (64/565, 11.3%). The broadcasts were equally positive, negative, or mixed, but with a higher neutral sentiment coefficient score. Doctors received less airtime (correlation of −0.14 with AIRTIME) as compared with nonexperts, but government experts received more airtime (correlation of 0.16 with AIRTIME). In general, featured guests showed more sentiments in their statements, with combined sentiment scores of 0.67: positive, negative, mixed, and neutral sentiments scored 0.16, 0.29, 0.21, and 0.33, respectively.

The results of the Tobit regression model estimation are shown in Table 3. There are 5 sets of columns, with the first column displaying the coefficient estimates of each variable and the second column displaying the *P* values. First, with respect to the DOCTOREXPERT variable, the coefficient estimate is negative and statistically significant (−0.181; *P*=.01); however, although its valence is primarily negative, its statistical significance is inconsistent across specifications, suggesting that other factors likely moderate the effect of DOCTOREXPERT on airtime.

**Table 3:** Full interaction model of Tobit regression results.^a,b^

Variables	DV^c^, airtime (seconds; log transformed)												
	1.1		1.2			1.3			1.4			1.5	
	All (SE)	*P* value	All (SE)	*P* value	All (SE)		*P* value	All (SE)		*P* value	All (SE)		*P* value
DOCTOREXPERT	−0.181 (0.070)	.01	−0.117 (0.085)	.17	0.147 (0.104)		.16	−0.197 (0.044)		<.001	−0.035 (0.191)		.86
GOVTEXPERT	0.674 (0.084)	<.001	0.281 (0.133)	.04	0.798 (0.054)		<.001	0.940 (0.561)		.09	0.612 (0.011)		<.001
POSITIVE	N/A^d^	N/A	0.267 (0.211)	.21	N/A		N/A	N/A		N/A	N/A		N/A
NEGATIVE	N/A	N/A	N/A	N/A	0.247 (0.057)		<.001	N/A		N/A	N/A		N/A
NEUTRAL	N/A	N/A	N/A	N/A	N/A		N/A	−0.723 (0.235)		<.001	N/A		N/A
MIXED	N/A	N/A	N/A	N/A	N/A		N/A	N/A		N/A	0.566 (0.266)		.03
DOCTOREXPERT × GOVTEXPERT	−0.631 (0.281)	.03	−0.667 (0.288)	.02	−0.873 (0.230)		<.001	−0.693 (0.353)		.05	−0.819 (0.356)		.02
DOCTOREXPERT × POSITIVE	N/A	N/A	0.286 (0.424)	.50	N/A		N/A	N/A		N/A	N/A		N/A
DOCTOREXPERT × NEGATIVE	N/A	N/A	N/A	N/A	−0.675 (0.148)		<.001	N/A		N/A	N/A		N/A
DOCTOREXPERT × NEUTRAL	N/A	N/A	N/A	N/A	N/A		N/A	0.344 (0.419)		.41	N/A		N/A
DOCTOREXPERT × MIXED	N/A	N/A	N/A	N/A	N/A		N/A	N/A		N/A	−0.225 (0.474)		.64
GOVTEXPERT × POSITIVE	N/A	N/A	0.938 (0.084)	<.001	N/A		N/A	N/A		N/A	N/A		N/A
GOVTEXPERT × NEGATIVE	N/A	N/A	N/A	N/A	−0.706 (0.356)		.05	N/A		N/A	N/A		N/A
GOVTEXPERT × NEUTRAL	N/A	N/A	N/A	N/A	N/A		N/A	−1.080 (1.162)		.35	N/A		N/A
GOVTEXPERT × MIXED	N/A	N/A	N/A	N/A	N/A		N/A	N/A		N/A	0.194 (0.831)		.82

^a^A set of control variables, including dummy variables for months and days, are included in the model.

^b^1.1: number of observations=564, log pseudolikelihood=−728.06, Akaike information criterion=1460.11; 1.2: number of observations=437, log pseudolikelihood=−536.41, Akaike information criterion=1076.82; 1.3: number of observations=437, log pseudolikelihood=−539.31, Akaike information criterion=1082.62; 1.4: number of observations=437, log pseudolikelihood=−533.23, Akaike information criterion=1070.47; 1.5: number of observations=437, log pseudolikelihood=−538.87, Akaike information criterion=1081.73.

^c^DV: dependent variable.

^d^N/A: not applicable.

Second, the GOVTEXPERT variable shows a positive coefficient and moderate to solid statistical significance across specifications, indicating that experts affiliated with the government received more airtime. Third, sentiments generally show positive coefficients compared with neutral sentiments. The positive (0.267; *P*=.21), negative (0.247; *P*<.001), and mixed (0.566; *P*=.03) sentiments are *positively associated* with airtime. However, neutral sentiment (−0.723; *P*<.001) is negatively associated with airtime. However, the coefficient for positive sentiment is not statistically significant, suggesting that a positive opinion may depend on other contextual factors.

The interaction term between DOCTOREXPERT and GOVTEXPERT is negative and statistically significant (−0.631; *P*=.03 for base model specification) across specifications, indicating that the 2 operationalized credibility variables amplify one another. Specifically, government-affiliated experts with a doctorate received less airtime compared with nonexperts.

More interestingly, we found that extreme valence sentiments, such as positive and negative sentiments, interact with the credibility variables for DOCTOREXPERT and GOVTEXPERT affiliation differently. For positive sentiments, there was a statistically significant interaction with GOVTEXPERT (0.938; *P*<.001) but not with DOCTOREXPERT (0.286; *P*=.50). For negative sentiments, we observed a significant interaction with both DOCTOREXPERT (−0.675; *P*<.001) and GOVTEXPERT (−0.706; *P*=.05), indicating that the relationship between expert affiliation and sentiment influences airtime differently depending on the valence of the sentiment.

These findings indicate that when experts express clear sentiments, it can directly impact the airtime they receive. Specifically, positive sentiments positively moderate the credibility of experts in gaining more airtime, whereas negative sentiments negatively moderate the credibility of experts in receiving less airtime than nonexperts. This suggests that the audience may be more interested in hearing positive news from authoritative sources and less interested in hearing negative news.

However, we found no statistical significance for neutral and mixed sentiments. This may suggest that regardless of credibility, neutral sentiments do not directly impact the amount of airtime received. One plausible explanation is that neutral sentiments may be perceived as uninteresting, and mixed sentiments may be perceived as confusing, resulting in less airtime dedicated to these sentiments.

### Robustness Checks

We checked the robustness of the Tobit regression results across the base models and with different interactions between the sentiment and experts’ relevant variables. The results remained relatively similar, with minor changes to the values of the coefficients.

We checked whether the results were affected by the software or procedure for coding the sentiment values. We acknowledge that our choice of AWSC to conduct sentiment analyses is based on a specific set of assumptions around the model. Nevertheless, we checked with Empath, VADER (Valence Aware Dictionary and Sentiment Reasoner), LIWC techniques, and AWSC tools for coding sentiment values. Empath and VADER are available as Python packages that rely on a lexicon-based approach to sentiment analysis using predefined dictionaries of words and phrases with assigned sentiment scores. VADER can handle negations and context-dependent sentiment classification and detect the intensity of emotions and sentiments; however, its accuracy may be lower than that of other methods. Our regression results, with the coded variables from Empath, VADER, and LIWC, remain similar, with some variations in the statistical significance. Broadly, we can say that LIWC and Empath are inconsistent because their sentiment methodology counts, but it does not adjust for the context, whereas results from AWSC and VADER both show consistent results.

We conducted a regression analysis using 3 sentiment polarity scores, whose values were predicted on a scale of 0 to 1. Unfortunately, the variational inflation factor on a simplified specification model consisting of all 3 sentiment polarities shows a variance inflation factor score above 2.5, a general index threshold for indicating multicollinearity, thereby limiting our ability to use the 3 sentiment dummies in the same models. We then merged the 3 categories into one variable, in which case the results came to be positive and showed significant interaction with DOCTOREXPERT and not significant with GOVTEXPERT variables. However, this does not indicate the positive or negative sentiment effects expressed in the comments.

### Additional Analyses

**Figure 1:**
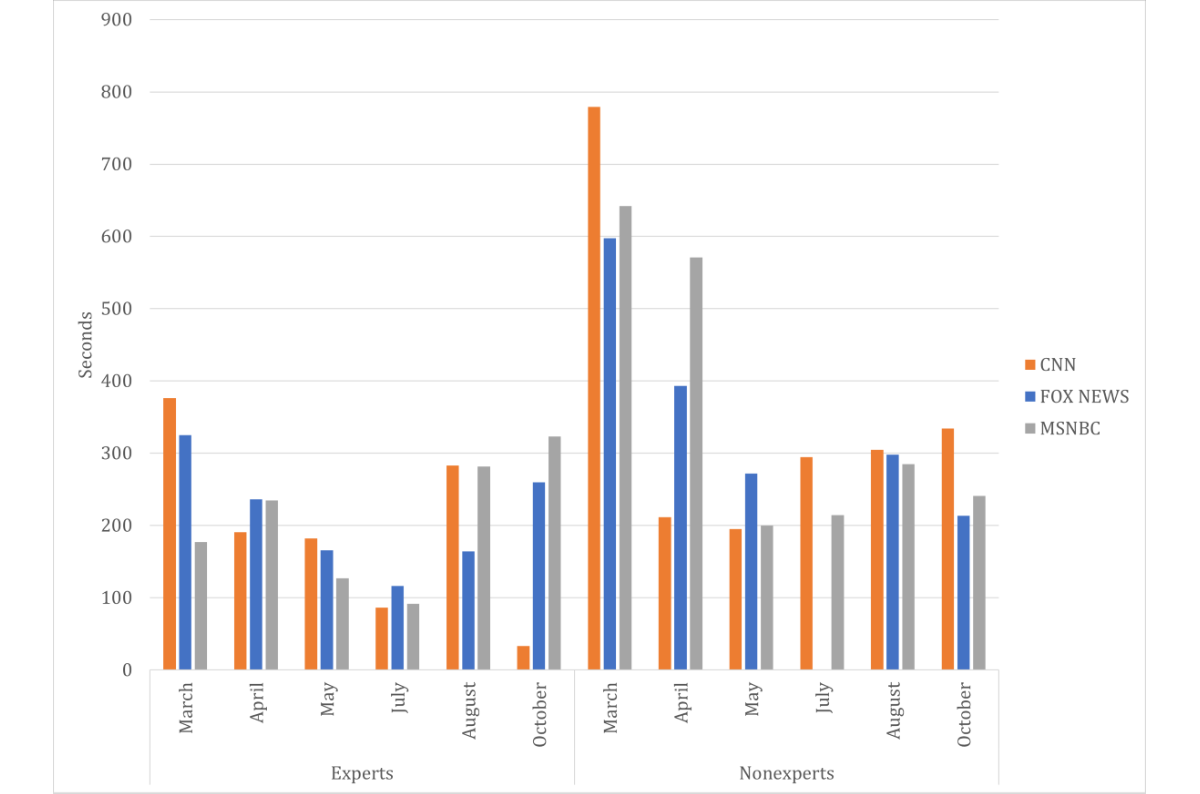
Comparison of experts versus nonexperts airtime across cable networks.

We conducted additional analyses to further delve into the details related to using expert sources in discussions about hydroxychloroquine as a therapeutic for COVID-19 on cable news networks and to understand any potential differences in using expert sources and messages among the networks. We found that nonexpert sources were used more frequently than expert sources to discuss the therapeutic validity of hydroxychloroquine and that the amount of airtime allocated to expert sources decreased over time (Figure 1).

We also found that a small number of experts accounted for a significant proportion of the total airtime allotted to experts on each network. The top 5 voices represented >40% of airtime on CNN and MSNBC and slightly >50% on FOX News (Table 4).

**Table 4:** Share of total airtime by featured experts across cable networks.

Name		Share (%)
**CNN^a^**		
	Sanjay Gupta	18.49
	Anthony Fauci	9.57
	Peter Hotez	4.89
	Celine Gounder	4.73
	Jonathan Reiner	4.51
**FOX News**		
	Mehmet Oz	23.02
	Deborah Birx	11.26
	Marc Siegel	6.89
	Nicole Saphier	5.51
	Stephen Hahn	5.2
**MSNBC**		
	Kavita Patel	15.78
	Amesh Adalja	8.43
	Natalie Azar	7.52
	Vin Gupta	6.64
	Ezekiel Emanuel	6

^a^CNN: Cable News Network.

The top 5 experts represented >40% of the airtime allotted to experts on CNN and MSNBC. On FOX News, the leading 5 experts accounted for slightly >50% of the airtime. An analysis of the top 3 experts shows some variability among the networks. The CNN medical correspondent Dr Sanjay Gupta received the most airtime, followed by Dr Fauci and Dr Peter Hotez, an expert in infectious diseases and vaccine development and dean of the National School of Tropical Medicine at Baylor College of Medicine.

**Figure 2:**
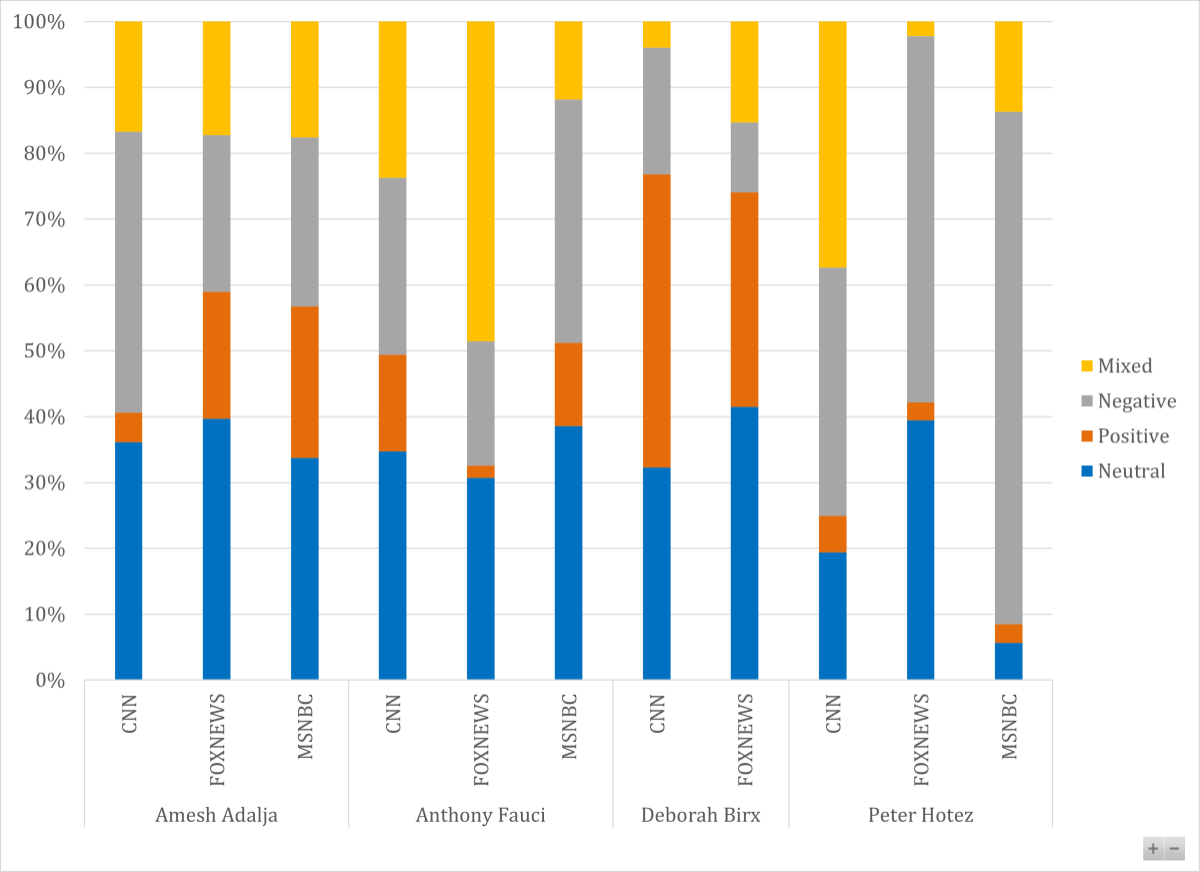
Comparison of sentiments across cable networks and top experts in sampled broadcasts. CNN: Cable News Network.

**Figure 3:**
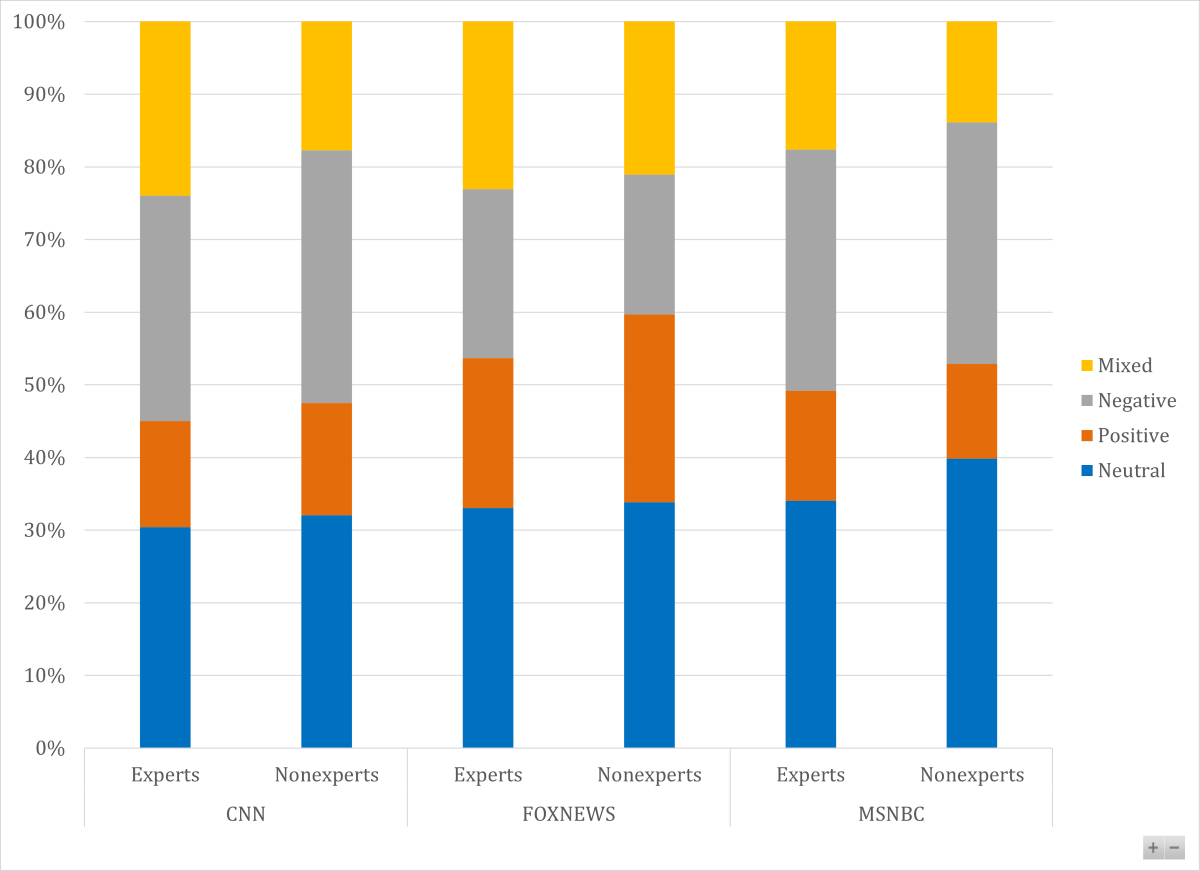
Comparison of sentiments across cable networks in sampled broadcasts. CNN: Cable News Network.

On FOX News, Dr Mehmet Oz, a celebrity, received the most airtime, followed by Dr Deborah Birx and Dr Marc Siegel, the FOX News medical correspondent. MSNBC does not have a dedicated medical correspondent. On MSNBC, Dr Kavita Patel, a former Federal Administration Official associated with the Center for Health Policy at the Brookings Institution, received the most airtime, followed by Dr Amesh Adalja, a senior scholar at the Johns Hopkins Center for Health Security, and Dr Natalie Azar, a National Broadcasting Company (NBC) News Medical Contributor and a professor at New York University Langone School of Medicine. Furthermore, our analysis revealed that the sentiment in the broadcast toward hydroxychloroquine was marked by a heated exchange of opinions and charged sentiments in contrast to a measured and thoughtful discussion. Both experts and nonexperts exhibited a range of sentiments, with positive, negative, and mixed sentiments occurring more frequently than neutral sentiments (Figures 2 and 3). Although some experts expressed negative views on the effectiveness of hydroxychloroquine and the negative consequences surrounding its use, others expressed positive sentiments and highlighted the ongoing studies on its potential use in the treatment and prevention of COVID-19. However, the experts emphasized the importance of studying the drug and informing the public.

## Discussion

### Explanation of Key Findings

Before discussing the implications of the findings of this study, we highlight the key findings. This study informs a substantial issue regarding how information is generated and disseminated to the public through cable television networks. Experts with advanced degrees, such as MDs and PhDs, are often seen as highly credible sources of information on public health issues. However, the findings suggest that they would receive less airtime on cable television. Academic expertise credibility may not be sufficient to be perceived as a persuasive dimension by the audience. Alternatively, these experts may not need much airtime except for what is taken to ratify the credibility. Government officials receive more time than nonexperts, even accounting for the positive or negative sentiments expressed during the broadcast. This could be due to their perceived authority from their positional power, confidence on camera, or experience with media appearances.

It is important to note that positive sentiments are generally associated with more airtime, whereas neutral or “boring” sentiments are negatively associated with airtime. Interestingly, displaying negative sentiments alone does not necessarily lead to less airtime. Instead, when doctors display negative sentiment, it impacts their airtime negatively. Mixed sentiments, in contrast, seem to be positively associated with airtime.

Thus, the findings raise concerns about accurate and comprehensive health information disseminated in cable television broadcasts. Given that academic experts do not get much airtime compared with government officials, does the public get a complete perspective? Should the media consider a balanced representation of credible sources to ensure the public gets accurate and comprehensive health information?

### Implications

The findings of this study have several practice and policy implications. First, this study draws insights into the influence of cable television on health communications and specifically highlights that broadcasters must be careful about the information they disseminate. Government officials get more airtime than academic experts, which may be because of their optimistic or biased statements. Academic experts who can provide more scientific facts do not get much airtime. Experts’ choices and preparations must be made carefully to instill credibility [12], a lack of which polarizes and politicizes health communications, which was evident during the pandemic [23,24].

This study sheds light on the specific context of information spread in cable broadcasting and its comparison with prior research on the spread of health information in social media [27]. Emotional-moral words correlate with a higher likelihood of being retweeted, and emotions are essential in making content contagious on social media [27-30]. Studies have shown how moral emotions shaped information spread on Twitter and other media about hydroxychloroquine as a solution to COVID-19 [29,30].

The findings have implications to highlight some elements of cable television discussions around hydroxychloroquine that differ from social media in 2 ways. First, a prior study has also identified and characterized scientific authority–related discussions about hydroxychloroquine in Twitter, alluding to medical experts’ credibility aspect of sources [27]. The findings of this study contrast with the earlier claim highlighting that there is less airtime for experts or authority figures in cable television broadcasting compared with the prominence of authority figures used on Twitter—a meaningful comparison as both authority figures and moral emotions shaped information spread on Twitter and other media about hydroxychloroquine as a solution to COVID-19 [29,30]. The contrast in the use of the expert’s credibility suggests that the medium of the discourse (cable vs Twitter) influences what type of content is spread or prominent; specifically, in the context of embedding the content with sentiments, both media have very different orientations for dissemination. These findings add further insights into how source credibility and health communications across different mediums differ in their shape, context, and ways of propagation.

Content broadcasts for scientific topics differ qualitatively from the information diffused through social media. The content broadcast on television is the product of a collaborative activity that includes scientists, journalists, editors, experts, and the public. The centerpiece of the collaboration is the interaction between journalists and their sources, often subject matter experts in their domain. The information provided by these experts helps shape and illuminate the story [36]. In the routine practice of science and medical journalism, journalists generally rely on material published in well-respected peer-reviewed journals, administrators of respected institutions, researchers, and sources that have previously spoken to the press [37]. The findings of this study raise a substantial challenge to public health communicators and specialists who are frequently advised to build working relationships with journalists [38]. However, different cable news networks may develop their relationships with different sources, and a few sources dominated the discussion about hydroxychloroquine.

Moreover, despite the available scientific data, or the lack of data in the initial stages, the expert sources on the different networks expressed different sentiments regarding its efficacy. Because viewers generally do not watch all 3 cable news networks, the information they received was dictated by the network they watch. For instance, FOX News viewers’ perspectives on the appropriateness of taking hydroxychloroquine differed sharply from CNN and MSNBC viewers. Particularly in the peak periods, when hydroxychloroquine was most frequently mentioned on cable news networks, the focus of the stories was not specifically on the merits or demerits of the drug. The expert opinions were expressed within the context of a broader newsworthy event. The experts also shared insights and discussed with nonexperts on the same broadcasts. The opinions of journalists, politicians, and others were often as prevalent as those of medical and scientific experts.

The mistaken suggestion that hydroxychloroquine could be used to treat COVID-19 had a real-world impact. Prescriptions written for the drug soared, resulting in thousands of people taking ineffective and potentially harmful treatment, which put pressure on the drug supply for those who needed it. The dynamics of the discussion about hydroxychloroquine are evidence of the development of filter bubbles and the polarization of critical public health information on cable news networks. They have an impact on decision-making and health outcomes. The divergences in outlook are not quickly addressed by typical health communication bromides, and public health officials should deliver consistent information in an appropriate format through channels of communication to which people attend. It requires different strategies to mitigate conflicting sentiments on complex public health issues.

Given the value of academic expertise to ensure that the public can access accurate and comprehensive health information, source credibility needs to be shown to the public in a way that they can assess and trust. This is a “trust-in-media” issue that goes beyond only viewership to be responsible for informing the public on significant health issues. We recommend that the channels indicate experts’ credibility during broadcasts. This will help the audience to reflect on the comments appropriately.

### Limitations and Directions for Further Research

This study has a few limitations that future studies may be able to address. First, the study’s data set focuses on the US viewership of cable news, focusing on the 3 major news networks. Therefore, our findings may not be generalizable to viewers elsewhere. Focusing on other issues around broadcasting may provide more nuanced and enriched explanations for the effect on the credibility-airtime associations. We did not capture everything in our models, and future studies may explore many such factors. This study was contextualized to the hydroxychloroquine-related discussions during the COVID-19 pandemic. The generalizability of other contexts remains a limitation that can only be explained after similar models have been applied to varied contexts in future studies. Another limitation of this study is that we used the cross-sectional data set to examine the relationships between variables. We believe that with multiple years of data from the same or similar contexts, future research will be able to provide causal inferences.

It could be argued that specific television programs, such as morning news or current affairs roundups, have prior agreements to allocate fixed interval times of airtime to featured guests to adhere to scripted formats. However, it becomes difficult to script and allocate a set time for individual experts regarding controversial topics such as hydroxychloroquine for COVID-19 as a therapeutic. In such cases, it is unlikely that experts are given predetermined amounts of airtime in a live, real-time show. Instead, the allocated airtime may depend more on their accessibility and the quality of the individual experts’ opinions [39]. For instance, the ability of experts to explain complicated information may increase allocated airtime. Alternatively, the depth or relevance of experts’ opinions may increase allocated airtimes. However, we acknowledge that the relationship between expert credibility and airtime allocation in this context does not necessarily indicate causality. A more robust analysis with other explanatory variables, experiments, or a panel data–oriented study design may be needed to establish causality. Thus, this study is exploratory and focuses on several controversial discussions held on major cable television channels in the United States regarding the role of hydroxychloroquine in the treatment of COVID-19. We used this unique and significant context to explore how the credibility of experts and the credibility of information influence the allocation of airtime in cable television.

### Conclusions

This study focused on the message that the credibility of broadcast sources is essential. These findings call for responsible behavior from broadcasters. The perceived credibility of the origin of the information is a critical determinant in guiding viewers’ evaluation of whether the information is true or false and consequently, the viewers’ opinion on the issue under discussion [40]. As was evident during the pandemic, discussions on the efficacy of hydroxychloroquine as a therapeutic for COVID-19 could potentially mislead the public into believing that there was a cure for COVID-19 that did not exist [25]. A cacophony of voices clamors for attention to any given topic, including politicians, journalists, and government officials. In the case of pandemics and other medical issues, doctors, scientific experts, and public discussions about hydroxychloroquine were no different [26]. Television channels need to be careful about health communications from experts.

## Abbreviations

AWSC: Amazon Web Service Comprehend
CNN: Cable News Network
LIWC: Linguistic Inquiry and Word Count
VADER: Valence Aware Dictionary and Sentiment Reasoner
